# Transcriptome Analysis Reveals Possible Immunomodulatory Activity Mechanism of *Chlorella* sp. Exopolysaccharides on RAW264.7 Macrophages

**DOI:** 10.3390/md19040217

**Published:** 2021-04-14

**Authors:** Siwei Wu, Hongquan Liu, Siyu Li, Han Sun, Xiumiao He, Ying Huang, Han Long

**Affiliations:** Guangxi Key Laboratory for Polysaccharide Materials and Modifications, School of Marine and Biotechnology, GuangXi University for Nationalities, Nanning 530006, China; wusiwei1125@outlook.com (S.W.); lsy15977732993@outlook.com (S.L.); sh18855092568@outlook.com (H.S.); xiumiaoe0839@sina.com (X.H.); hying946965@outlook.com (Y.H.); gu0ling@live.com (H.L.)

**Keywords:** *Chlorella*, polysaccharide, immune activity, immune mechanism, transcriptome

## Abstract

In this study, the exopolysaccharides of *Chlorella* sp. (CEP) were isolated to obtain the purified fraction CEP4. Characterization results showed that CEP4 was a sulfated heteropolysaccharide. The main monosaccharide components of CEP4 are glucosamine hydrochloride (40.8%) and glucuronic acid (21.0%). The impact of CEP4 on the immune activity of RAW264.7 macrophage cytokines was detected, and the results showed that CEP4 induced the production of nitric oxide (NO), TNF-α, and IL-6 in a dose-dependent pattern within a range of 6 μg/mL. A total of 4824 differentially expressed genes (DEGs) were obtained from the results of RNA-seq. Gene enrichment analysis showed that immune-related genes such as *NFKB1*, *IL-6*, and *IL-1β* were significantly upregulated, while the genes *RIPK1* and *TLR4* were significantly downregulated. KEGG pathway enrichment analysis showed that DEGs were significantly enriched in immune-related biological processes, including toll-like receptor (TLR) signaling pathway, cytosolic DNA-sensing pathway, and C-type lectin receptor signaling pathway. Protein–protein interaction (PPI) network analysis showed that *HSP90AB1*, *Rbx1*, *ISG15*, *Psmb6*, *Psmb3*, *Psmb8*, *PSMA7*, *Polr2f*, *Rpsa*, and *NEDD8* were the hub genes with an essential role in the immune activity of CEP4. The preliminary results of the present study revealed the potential mechanism of CEP4 in the immune regulation of RAW264.7 macrophages, suggesting that CEP4 is a promising immunoregulatory agent.

## 1. Introduction

Immune system disorders can affect the immune response of the body and cause immune diseases. It has become increasingly important to use immunomodulators to treat and prevent human diseases caused by the destruction of the immune system [1,2]. Over the years, polysaccharides from natural sources have attracted increasing attention due to their antitumor, immunomodulatory activities, and low toxicity. Polysaccharides from plants [3], bacteria [4,5] and fungi [6] have been reported to have immunoregulatory abilities.

Many microalgae polysaccharides have exhibited immunomodulatory abilities [7,8,9,10]. In recent years, bioactive natural products extracted from microalgae have become a research hotspot; their extracellular products have particularly attracted significant attention. It has been reported that polysaccharides from the green microalga *Chlorella* have a variety of biological activities, such as anti-asthma [11], antioxidation [12], blood lipid-decreasing capacity [13], anticancer, and immunoregulatory activities [14]. Studies have shown that polysaccharides from *Cyanobacteria spirulina* can enhance the immune function in mice [15,16]. Qi et al. extracted the intracellular polysaccharides from *Chloroidium ellipsoidea* and studied its potential immune mechanism on macrophages [17]. However, the immunomodulatory activity mechanism of extracellular polysaccharides from *Chlorella* remains to be elucidated.

To clarify the immunomodulatory activity mechanism of CEP, the impact of CEP4 on the immune activity of RAW264.7 macrophage cytokines was detected, and the relationship between immune activity and related gene expression was studied through transcriptomics analysis to develop an experimental basis for using CEP in treating human diseases caused by immune system compromise.

## 2. Results

### 2.1. Isolation and Purification of Crude CEP

**Figure 1:**
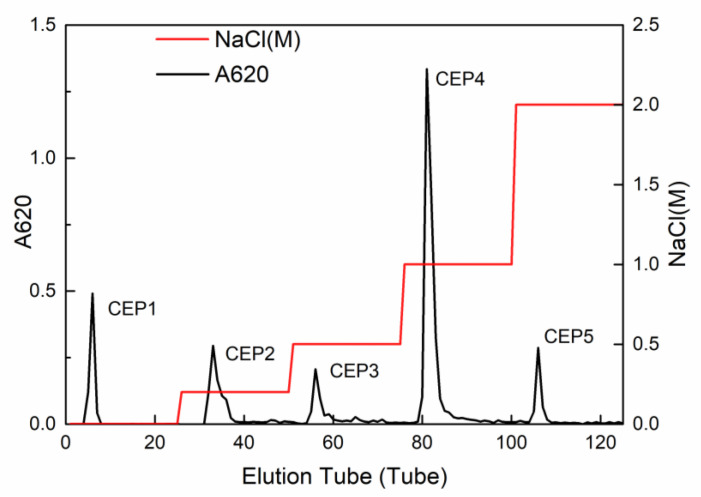
The elution profile of crude polysaccharide from *Chlorella* on DEAE-cellulose anion-exchange chromatography column.

The crude CEP was purified by DEAE-cellulose (DEAE-52) anion-exchange chromatography column, and five main independent peaks, namely CEP1 (eluted by ultrapure water), CEP2 (eluted by 0.2 M NaCl), CEP3 (eluted by 0.5 M NaCl), CEP4 (eluted by 1 M NaCl), and CEP5 (eluted by 2 M NaCl), were obtained (Figure 1). This study mainly focused on CEP4, and the other fractions will be studied in future research.

### 2.2. Characterization of CEP4

**Figure 2:**
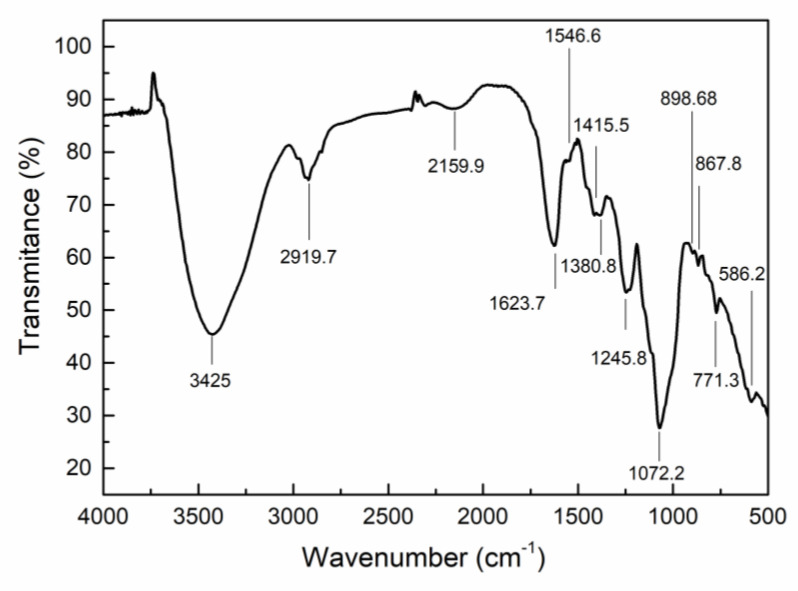
FT-IR spectrum of CEP4 from *Chlorella* sp.

Figure 2 shows the FT-IR spectrum. A wide and intense absorption peak at 3425 cm^−1^ and a small signal at 2919 cm^−1^ are the characteristics of the stretching vibration of –OH and C–H, respectively [18,19]. Meanwhile, FT-IR bands at 1623 and 1415 cm^−1^ arise from asymmetric and symmetric stretching vibrations of carboxylate [20,21]. Consistent with other sulfated polysaccharides, the peaks were recorded at 1245 and 867 cm^−1^, corresponding to stretching vibration of S–O and bending vibration of C–O–S of sulfate [22]. More importantly, the band at 898 cm^−1^ is characteristic of β-polysaccharide [20]. Overall, the FT-IR results indicated that CEP4 is a sulfated polysaccharide.

**Figure 3:**
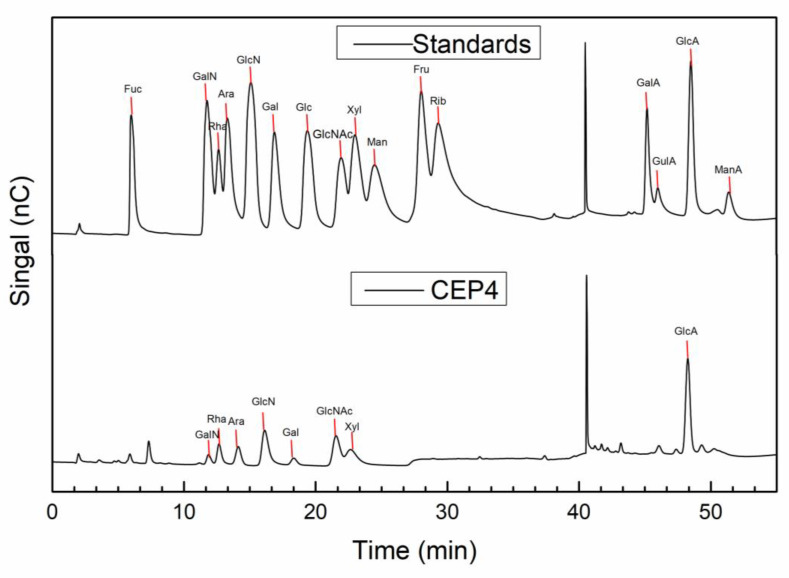
IC chromatograms of monosaccharide composition of CEP4.

Based on the IC analysis (Table 1 and Figure 3), the monosaccharide composition of CEP4 was determined to be GlcN (40.8%), GlcA (21%), Xyl (8.6%), Ara (8.3%), GlcNAc (7.7%), Rha (3.7%), and Gal (2.6%). The results suggested that CEP4 is a heteropolysaccharide mainly consist of glucose and glucuronic acid.

### 2.3. Effects of Polysaccharide on the Activation of the Innate Immune Response In Vitro

#### 2.3.1. Effects of Polysaccharides on NO Production

**Figure 4:**
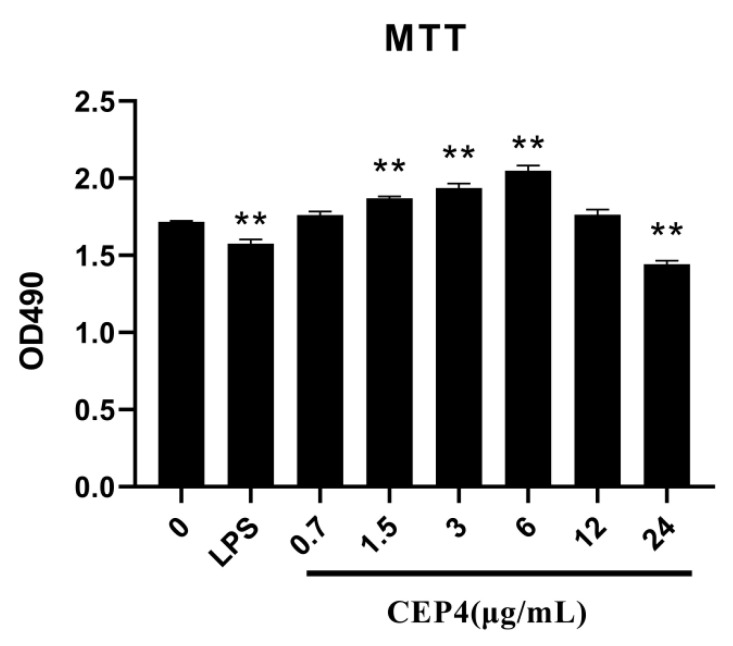
The toxicity of CEP4 on RAW264.7 macrophages. ** (*p* < 0.01) represent significant difference against untreated group. Data are represented as the mean ± SD of at least three independent experiments (three replicates in each time).

A high value of OD490 indicated that CEP4 had a promoting effect on the survival of macrophages, while a low value of OD490 indicates that CEP4 has a toxic effect on macrophages. In Figure 4, CEP4 showed no cytotoxicity on RAW264.7 macrophages at 6 μg/mL. 

**Figure 5:**
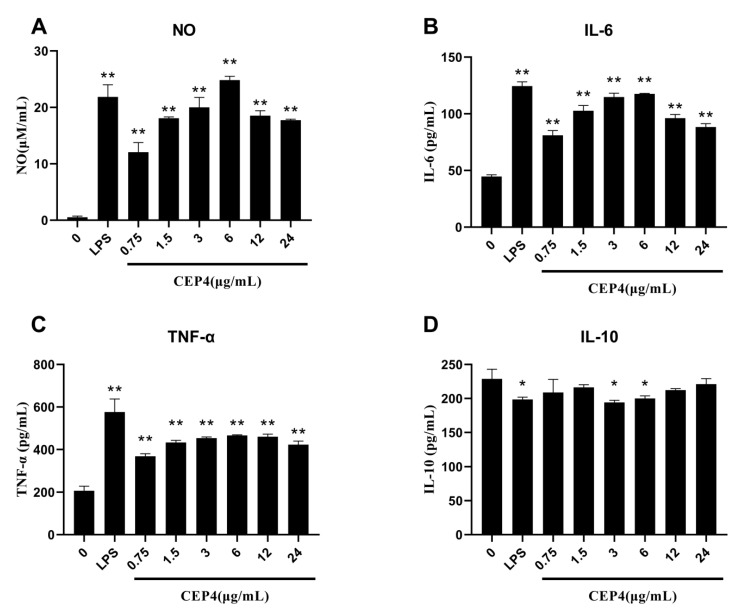
NO and cytokine production of RAW264.7 macrophage cells after treatment with different concentrations of CEP4. Panels (**A**–**D**) correspond to the contents of NO, IL-6, TNF-α, and IL-10 secreted by RAW264.7 macrophage cells, respectively. * (*p* < 0.05), ** (*p* < 0.01) represent significant difference against untreated group. Data are represented as the mean ± SD of at least three independent experiments (three replicates for each time).

The level of NO production could be used as a quantitative index of macrophage activation [22]. The RAW264.7 macrophages stimulated by LPS are a mature model for studying the inflammatory response [23]. The Griess method was used to detect the production of NO in macrophage supernatant to evaluate the immunostimulatory activity of CEP4. The result showed that the NO concentration significantly increased, nearly 20 times, compared with the control group (Figure 5A). At lower concentration range, the secretion of NO increased with an increase in CEP4 concentration, while higher concentrations did not promote NO release.

#### 2.3.2. Effects of Polysaccharides on Cytokines 

After confirming that CEP4 could induce macrophages to release NO, the IL-6, TNF-α, and IL-10 concentrations were detected by ELISA. At 6 μg/mL, CEP4 significantly increased IL-6 production in a dose-dependent pattern and began to saturate when the concentration increased above 6 μg/mL (Figure 5B). However, a high saturation rate reduced the release of IL-6 because very high concentrations of CEP4 might have inhibited the activity of cells exerting toxic effects on macrophages. Like IL-6, the release of TNF-α also increased significantly in the same trend but did not reach the release level of LPS group (Figure 5C). It is established that IL-10 is an anti-inflammatory cytokine. Similar to the IL-10 trend in the LPS model group, the release of IL-10 from macrophages treated with CEP4 decreased, indicating that its effect might be consistent with the effect of LPS. This finding indicates that CEP4 exhibits a pro-inflammatory activity. In addition, compared with the control group, we also found that 3 and 6 μg/mL concentration of CEP4 showed significant differences (Figure 5D) closer to LPS model.

### 2.4. Results of RNA-seq

#### 2.4.1. Analysis of DEGs

**Figure 6:**
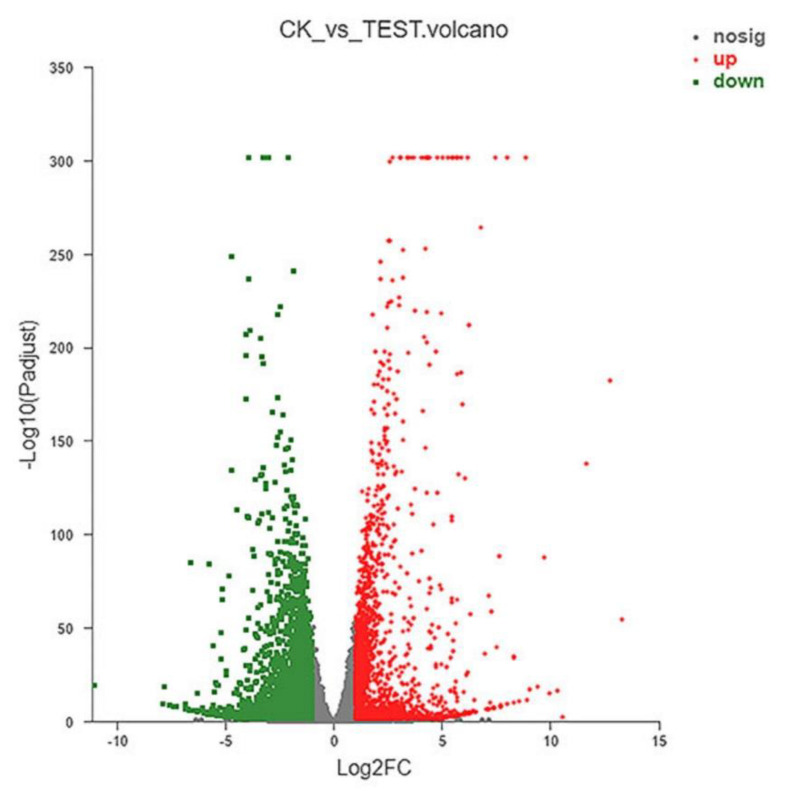
Visual analysis of DEGs. Scatter diagram of DEGs between CK and Test group. The red dots indicate that genes were significantly upregulated, green dots indicate that genes were significantly downregulated, and gray dots indicate that genes were not significantly different.

More than 44 million clean reads were generated for each CK and test group, with which the rate of total mapping was >90.98%. Differential expression analysis was performed using the DESeq2 R package in the CK and test groups and a volcano plot of DEGs was constructed (Figure 6). The red dots represent significantly upregulated genes (n = 2161), with the green dots representing significantly downregulated genes (n = 2659). The function of these genes is mainly related to energy production and conversion, signal transduction mechanisms, RNA function, cell cycle control, chromatin structure and dynamics, transport and metabolism, and defense mechanisms.

#### 2.4.2. GO Enrichment of DEGs

**Figure 7:**
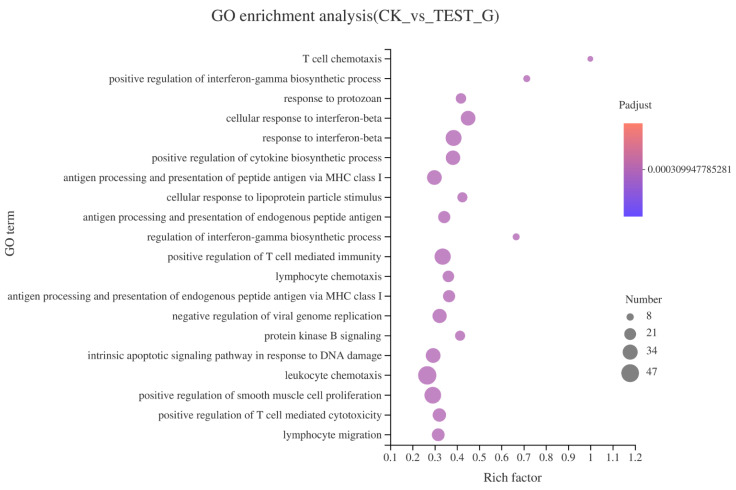
Bubble diagram of GO enrichment of DEGs. The x-axis represents the Rich factor, and the y-axis represents the GO terms. The higher Rich factor represent the greater degree of enrichment in the GO term, the size of the dot represents the number of genes in the GO Term, and the color of the dot corresponds to different *P*-adjust ranges.

To further understand the GO function of differentially expressed genes (DEGs), GO enrichment analysis (*p* < 0.05) was performed. Figure 7 shows the top 20 significant enriched GO terms for the DEGs. The results showed that T cell chemotaxis occupied the highest enrichment rate as it possessed the highest rich factor (1), followed by positive regulation of interferon-gamma biosynthetic process (rich factor 0.714). GO enrichment analysis showed that T cell chemotaxis, positive regulation of interferon-gamma biosynthetic process, regulation of interferon-gamma biosynthetic process, cellular response to interferon-beta, and cellular response to lipoprotein particle stimulus were the main types in biological process (BP), indicating that CEP4 had the most significant effect on the BP of RAW264.7 macrophages. Most DEGs involved in these processes, such as *Ccr2*, *Ccl3*, *CxCr3*, *Gpr183*, *Il27*, and *Tlr7*, were related to the immune response. 

#### 2.4.3. KEGG Pathway Enrichment of DEGs 

**Figure 8:**
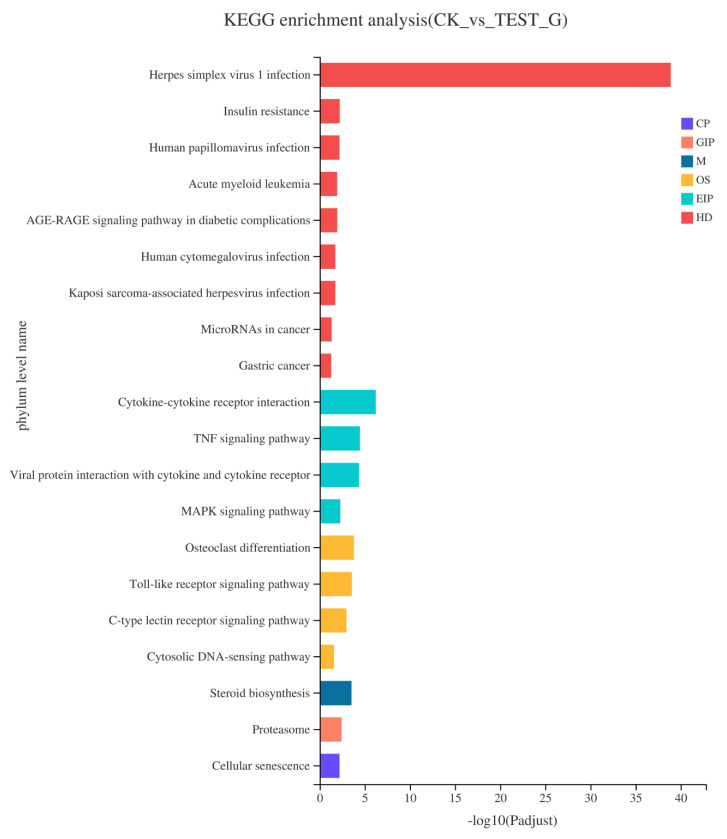
The histogram of KEGG pathway enrichment analysis of DEGs. The x-axis represents the significance level of enrichment, and the y-axis represents the KEGG pathway which corresponds to the height of the column. Among them, the smaller the FDR and the larger the −log10 (FDR) value represents the more significant enrichment of this KEGG pathway.

KEGG analysis was performed to identify the gene associated with the activation of macrophage immune activity by CEP4. In the CK and Test groups, 19 metabolic pathways (Figure 8), mainly distributed in four branches of the KEGG pathway, were significantly enriched. Among them, there were eight human diseases (HD), four environmental information processing (EIP), four biological systems (OS), one metabolic (M), one genetic information processing (GIP), and one cellular process (CP). Furthermore, among the 19 significantly enriched pathways, DEGs were significantly enriched in many signaling pathways, including TNF signaling pathway, MAPK signaling pathway, toll-like receptor signaling pathway, C-type lectin receptor signaling pathway, and cytosolic DNA-sensing pathway. DEGs related to the immune system were enriched in the toll-like receptor signaling pathway, cytosolic DNA-sensing pathway, and C-type lectin receptor signaling pathway, indicating that differences in DEGs between the CK and CEP4 groups were closely related to immune signaling pathways. This is not only helpful for us to investigate the effect of CEP4 on RAW264.7 macrophages but also provides vital information for further analysis.

#### 2.4.4. PPI Network Analysis

**Figure 9:**
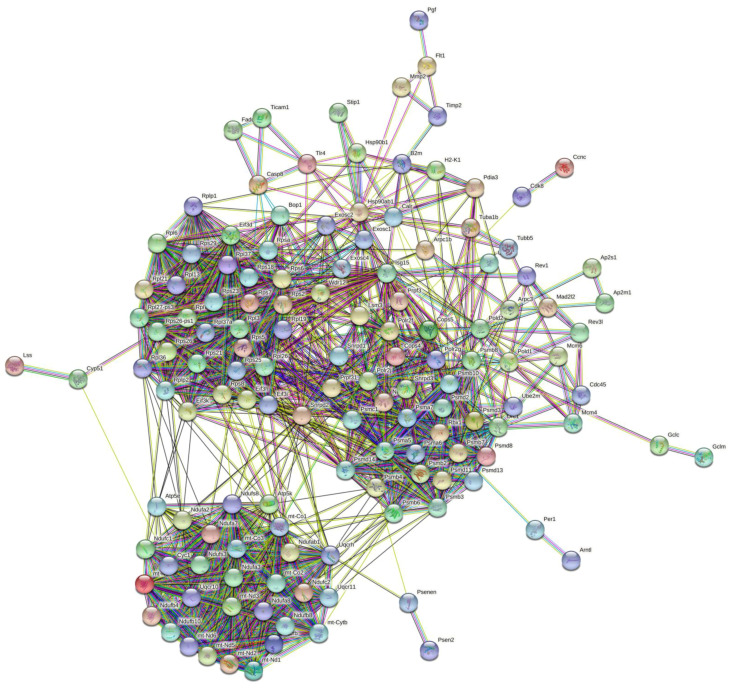
STRING interaction Network and PPI network of DEGs and the expression patterns of 8 DEGs determined by qPCR. The network nodes represent the protein of DEGs; lines indicate association between the linked DEGs.

**Figure 10:**
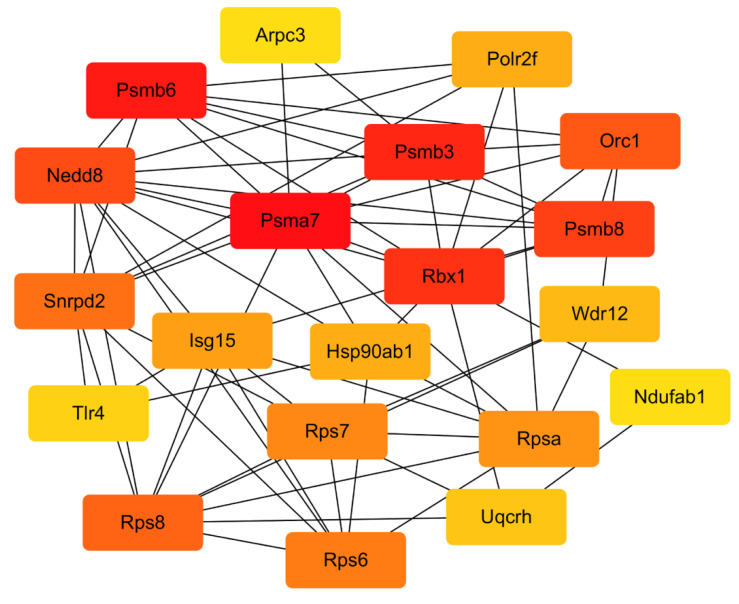
The 20 genes associate with immunity as calculated by Betweenness.

DEGs were mapped into the STRING database for the PPI network to explore the interactive relationship between DEGs. Figure 9 shows that the STRING interaction network consisted of 137 nodes, and the top 10 genes were obtained by the Betweenness algorithm by Cytohubba plug-in in the Cytoscape software (Table 2, Figure 10). These genes have a strong connection with other genes, indicating that they are the hub genes in the PPI network and might play an essential role in the activation of macrophages by CEP4. 

#### 2.4.5. qPCR Validation of DEGs

**Figure 11:**
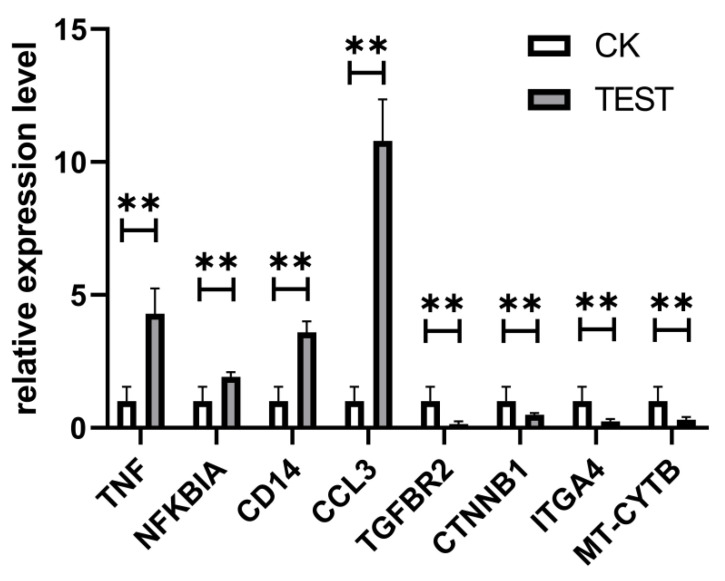
qPCR validation of DEGs. RAW264.7 cells were treated with CEP4 for 24 h, the total RNA extracted, and the expression of 8 randomly selected DEGs was determined by qPCR. Data are represented as the mean ± SD of at least three independent experiments. ** (*p* < 0.01) represent significant difference against untreated group.

To validate the RNA-seq, several DEGs were randomly selected for qPCR. As shown in Figure 11, the expression of genes *Tnf*, *NFKB*, *Cd14*, and *Ccl3* were significantly upregulated while *TGFBR2*, *CTNB1*, *ITGA4*, and *MT-CYTB* were significantly downregulated. Taken together, the pattern and extent of gene expression determined by the qPCR analysis were consistent with the RNA-seq analysis, confirming the agreement between these two methods and validating our RNA-seq-based gene expression analysis.

## 3. Discussion

Screening new natural compounds that enhance immune responses has become an important research direction in immunopathology and tumor therapy. To search for new biologically active compounds, we isolated, purified, and characterized the extracellular polysaccharide from *Chlorella* sp. and studied the immune activity and mechanism of CEP4. Characterization results showed that the monosaccharide components of CEP4 are glucosamine hydrochloride and glucuronic acid. Qi et al. extracted intercellular polysaccharides with immuno-enhancement function from *Chlorella ellipsoidea*, consisting of glucose and galactose [17]. These indicate that the compositions of *Chlorella* polysaccharides from different sources are different. 

By playing an essential role in regulating innate and adaptive immunity [24], macrophages are essential for maintaining body homeostasis and resisting foreign pathogens by producing inflammatory mediators [25]. ELISA experiments showed that at low concentrations, CEP4 stimulated macrophages to produce pro-inflammatory cytokines TNF-α and IL-6 in a concentration-dependent pattern. Cytokines are small molecular polypeptides and glycoproteins that mediate intercellular interactions. TNF-α is a cytokine produced by macrophages, with a role in the inflammatory response, cellular immunity, tumor immunity, and other physiological processes, while IL-6 is a pro-inflammatory cytokine that stimulates the immune system to repair damaged cells and promote the host survival [26,27]. Many polysaccharides from algae have demonstrated immunoregulatory activity. Sulfated polysaccharides from red seaweed *Nemalion elminthoides* exhibited immunomodulatory activity in vitro and in vivo [28]. On the other hand, sulfated polysaccharides from the filamentous microalgae *Tribonema* sp. can significantly stimulate and enhance the immune activity of macrophages and release the anti-inflammatory cytokine IL-10 to improve the excessive activation of macrophages [29]. The present study showed that CEP4 reduced the release of IL-10 from RAW264.7 macrophages, consistent with the LPS model, indicating that the immunomodulatory effect of CEP4 on macrophages is mediated by the secretion of TNF-α and IL-6. However, polysaccharides from the green microalga *Dunaliella salina* demonstrated an immune stimulatory effect on PBMC cells, with an anti-inflammatory activity on macrophages, indicating that microalgae polysaccharides from different sources might have different immune regulatory effects on macrophages [30]. NO, which is usually used as an immune indicator, plays a vital role in eliminating dissidents, invading pathogenic bacteria, tumor cells, and inflammatory damage [31,32]. It can also quickly transmit the cell’s information as it is a cellular messenger. Similarly, CEP4 can also significantly induce NO production in macrophages. In conclusion, these findings indicate that CEP4 is a potentially effective immunomodulator.

RNA-seq can accurately reveal the interaction between CEP4 and macrophages and changes in the whole signaling pathway of macrophages after treating with CEP4. A total of 4824 DEGs were screened from the analysis of RNA-seq, including 2165 upregulated and 2659 downregulated DEGs. The significant enrichment pathways related to immunity are toll-like receptor signaling pathway, C-type lectin receptor signaling pathway, and DNA-sensing pathway. It has been reported that RAW264.7 macrophages produce inflammatory mediators through NF-κB and MAPK signaling pathways [33,34]. NF-κB protein is a significant transcriptional regulator that regulates the transcription of immune-related genes in macrophages and plays a crucial role in inflammation, immunity, and cell proliferation [35,36]. MAPK can regulate gene expression, immune response, cell growth, differentiation, apoptosis, and other processes [37]. KEGG enrichment results showed that the expression levels of *NFKB1*, *TNF*, *Fas*, and *IL-1* genes that are closely related to immune function significantly increased in the MAPK signaling pathway. RIPK1 kinase, which can activate the NF-κB signaling pathway required for inflammatory changes in macrophages, can control the expression of inflammatory genes induced by LPS [38]. Joanne reported that the increased inflammation caused by *RIPK1* specifically depends on the TLR signal of the MyD88 pathway [39]. Previous studies have shown that the main receptors to recognize polysaccharides in macrophages are TLR4, CD14, complement receptor type 3 (CR3), and scavenger receptor (SR) [40]. TLR is a pathogen-associated molecular pattern (PAMP) sensor that plays an essential role in immune response [41]. When TLR4 binds to LPS, it motivates the MyD88-mediated signaling cascade, activating the transcription factor NF-κB and ultimately causing an inflammatory response [42,43]. It has been demonstrated that the intracellular polysaccharides from *Auxenochlorella pyrenoidosa* proteinase activate the immune activity of macrophages via the TLR4 receptor [44]. CEP4 aggravates the production of inflammatory factors and induces pro-inflammatory responses through the downregulation of *TLR4* and *TLR6* and the upregulation of *CD14* in the toll-like pathway. In significantly enriched pathways, it seems the upregulation of *TNF* is mediated by NF-κB. However, a review of *TNF* gene transcriptional regulation shows that it has no clear functional role for NF-κB motifs in the activation of murine *TNF* gene transcription [45]. Further studies are necessary on the relationship between *TNF* expression and NF-κB in macrophages treated with CEP4. Overall, these data suggest that polysaccharides aggravate the inflammatory response by upregulating *NFKB1* and *IL-1β* genes and downregulating *RIPK1*, *TLR4*, and *TLR6* genes.

A series of hub genes, including *Hsp90ab1*, *Rbx1*, *Isg15*, *Snrpd2*, *Polr2f*, *Uqcrh*, *Psmb8*, *Psmb3*, *Psmb6*, *Rpsa*, *Orc1*, and *NEDD8*, were obtained from the analysis of the PPI network, most of which are related to immunoregulation. *HSP90* family genes are associated with a variety of cellular pathways [46]. *HSP90AB1*, one of the *HSP90* family genes, has a role in innate immunity [47]. It is distributed and expressed in immune cells infected with pathogens, promoting the invasion and metastasis of cancer cells through the synergistic effect of PI3K and MAPK signaling pathways. *RPSA* is a multifunctional and highly conserved ribosomal protein widely expressed in various cells [48,49]. *Rbx1* is a member of the E3 ubiquitin ligase family, mainly present in the nucleus and cytoplasm [50]. Siyuan Ding found that siRNA knockdown and chemical inhibition of the activity of *Rbx1* could suppress the degradation of β-TrCP mediated by RV encoding non-structural protein 1 (NSP1), which was beneficial to the activation of NF-κB signaling pathway, effectively reducing the ability of NSP1 to mount an antagonistic host immune response and inhibiting virus reproduction [51]. *Rbx1* is also associated with the antitumor DMC that can improve the immune microenvironment of hepatocellular carcinoma cells [52]. *ISG15*, which was wildly studied in immune processes, is a 15 kDa ubiquitin type I IFN that stimulates proteins and has various functions, such as activating immune cells, inducing the secretion of cytokines, and acting as a mediator of the immune response [53,54,55]. On the other hand, *Psmb3*, *Psmb6*, and *Psmb8* are the members of the proteasome subunit and have been found in most cancers [56]. *Psmb6* is a constituent proteasome, while *Psmb7* and *Psmb8* are immune proteasomes, and both play an important role in MHC Class I molecular antigen presentation [57,58,59]. Serving as an immune regulator, the proteasome *PSMA7* could negatively regulate mitochondrial antiviral signaling (MAVS), mediating innate immune response and effectively inhibiting RIG-1 and MAVS-mediated IFN-β promoter activity [60]. On the other hand, the hub gene *Polr2f* is often found in cancer cells [61,62]. The NEDD8-activating enzyme, which mediates an immune effect, can be used as a tractable target in chronic lymphocytic leukemia (CLL) and non-Hodgkin lymphoma [63]. However, there is no direct evidence on the relationship between CEP4 and these hub genes. Therefore, further studies are necessary to investigate the effort of CEP4 and hub genes on immunoregulation.

## 4. Materials and Methods

### 4.1. Materials and Chemicals

*Chlorella* sp. used in the experiments was purchased from Shanghai Guangyu Biological Technology Co., Ltd. (Shanghai, China) and bred by mutagenesis in our laboratory. The RAW264.7 macrophage cell line was preserved in our laboratory. DEAE-cellulose (DE-52) was purchased from Shanghai Rui Yong Biotechnology Co., Ltd. (Shanghai, China). The detection kit for NO was purchased from Nanjing Jiancheng Bioengineering Institute (Nanjing, China) and the ELISA kit for TNF-α, IL-6, and IL-10 was purchased from Shanghai Jining Industrial Co., Ltd. (Shanghai, China). FBS, DMEM, and PBS used for cell culture were all purchased from the Biological Industries.

### 4.2. Extraction of Crude CEP 

*Chlorella* sp. was cultivated up to the stable period, and the supernatant was obtained by centrifugation which was concentrated by a rotary evaporator and dialyzed using a dialysis bag. The protein was removed and precipitated with 4 volumes of absolute ethanol using Sevage reagent. The crude *Chlorella* sp. extracellular polysaccharide was obtained by vacuum freeze-drying.

### 4.3. Isolation and Purification of Crude CEP 

DEAE-cellulose (DE-52) anion-exchange chromatography column was applied to separate the crude CEP [64]. The CEP precipitate was dissolved by ultrapure water filtered with an 0.22 μm microporous membrane and then loaded on DEAE-cellulose (DE-52) anion-exchange chromatography column. Subsequently, the column was eluted with different concentrations of NaCl (0, 0.2, 0.5, 1, and 2 M). Five types of fractions (8 mL per tube), labeled as CEP1, CEP2, CEP3, CEP4, and CEP5, were separately collected and the total carbohydrate contents quantified using the anthrone sulfuric acid method.

### 4.4. Characterization of CEP4

#### 4.4.1. FT-IR Spectroscopy 

CEP4 was mixed with KBr (1% *w*/*w*), and IR spectra of the mixture were collected on a Thermo Nicolet Avatar 360 IR spectrophotometer in the range of 4000–500 cm^−1^ by using the KBr pressed-disk method.

#### 4.4.2. Monosaccharide Composition of CEP4

After hydrolyzing with 3 M trifluoroacetic acid at 120 °C for 3 h, the monosaccharide composition of CEP4 was determined by ion chromatography (IC) (ICS5000, Thermo Fisher). IC was performed in a Dionex CarbopacTM PA20 column (3 × 150) at 30 °C, eluting with H_2_O, 250 mM NaOH, 50 mM NaOH, and 500 mM NaOAC, in turn, at a flow rate of 0.3 mL/min. An electrochemical detector (ECD) was used for detection. 

### 4.5. Cell Culture Immune Activity of CEP4

#### 4.5.1. Cell Viability Assay 

The murine macrophage cell line RAW264.7 was incubated at 37 °C in a 5% CO_2_ environment and propagated in DMEM (high glucose medium with 10% heat-inactivated FBS, 100 U/mL of penicillin, and 100 mg/mL of streptomycin).

The MTT assay was used to measure the viability of cells. RAW264.7 macrophage cells (1 × 10^5^ cell/well) were plated in 96-well plates for 12 h. The medium was discarded, followed by washing with PBS and treating with different concentrations of CEP4 (0.75, 1.5, 3, 6, 12, and 24 μg/mL) for 24 h. The cells treated with an equal volume of medium without CEP4 were used as the negative control while the cells treated with the medium of lipopolysaccharides (LPS) (1 μg/mL) were used as the positive control [65]. After treatment, 10 μL of MTT (5 mg/mL), which was dissolved in PBS, was added to each well and incubated for another 4 h. Then, the medium was discarded, and the formazan crystals present in cells was dissolved by DMSO. The absorbance of the solution was measured at 490 nm in a microplate reader. The results were expressed in terms of absorbance, and there were two replicates for each treatment. 

#### 4.5.2. Determination of Nitric Oxide

Similar to a previous study, NO production was monitored by the accumulation of nitrite [66]. The cells (1 × 10^5^ cells/well) were seeded in a 24-well cell plate and incubated as mentioned above. After the treatment with CEP4, the supernatants of the cell cultures were collected by centrifugation and used for NO detection. The production of NO was estimated by the Griess reagent kit according to the manufacturer’s instructions. The untreated and LPS groups were used as controls.

#### 4.5.3. Secretion of Cytokines

The RAW264.7 cells were cultured and treated with different concentration of the CEP4 for 24 h. The TNF-α, IL-6, and IL-10 levels were determined with ELISA kits according to the manufacturer’s protocol. 

### 4.6. Transcriptome Analysis

#### 4.6.1. RNA Extraction, Library Construction, and Sequencing

RAW264.7 cells were treated with 6 μg/mL CEP4 for 24 h. TRIZOL was added to fully dissolve the cells and extract the total RNA according to the manufacture protocol. The following procedures such as RNA quality inspection, library construction, and sequencing, were assigned to Shanghai Meiji Biotechnology Co., Ltd. (Shanghai, China).

#### 4.6.2. Sequence Quality Control and Data Processing

Clean reads were obtained using Sickle and Seqprep software to remove low quality reads, including reads containing adapter and poly-N stretches and nucleotides with a quality score < 20. Before reconstructing the transcriptome conducted by software cufflinks, clean reads were mapped to the reference genome using HISAT 2.

#### 4.6.3. Analysis of DEGs 

A *P*-adjust of <0.05, a false discovery rate (FDR) of <0.01, and an estimated absolute log2-fold change of >0.05 were selected as the threshold to determine the significant differentially expressed gene in the libraries. RSEM program was applied for quantifying the frequency of the gene while the transcripts per million (TPM) reads were performed to normalize the level of gene expression.

#### 4.6.4. Gene Ontology (GO) and Kyoto Encyclopedia of Genes and Genomes (KEGG) Enrichment

DEGs were further analyzed by GO enrichment and KEGG pathway enrichment [67]. DEGs were mapped to the GO database (http://www.geneontology.org/, accessed on 25 February 2021) for GO enrichment analysis using Goatools software, while the pathway enrichment analysis was carried out using Kyoto Encyclopedia of Genes and Genomes (http://www.genome.jp/kegg/, accessed on 25 February 2021). It was considered significantly enriched in DEGs when the corrected *p*-value (FDR) was <0.05.

#### 4.6.5. Analysis of PPI Network and Hub Genes 

The PPI network, constructed using the online STRING database (http://string-db.org/, accessed on 25 February 2021), was used to investigate the potential relationship between DEGs and a condition with a total score of >0.7. Cytoscape software (version 3.7.2) was used to visualize the PPI network, while the Cytohubba plug-in was employed to calculate the Node’s score with the Betweenness algorithm [68].

### 4.7. qPCR Analysis of DEGs

Several DEGs were selected randomly for qPCR validation based on the results of RNA-seq to verify the accuracy and confidence of CEP4 treatment affecting the differential gene expression level in RAW264.7 macrophages. First, the single-strand cDNA which can directly be applied as a template was synthesized from mRNA, prepared in the above, for RNA-seq by 5× All-In-One RT MasterMix (Cat.No. G492, Applied Biological Materials Inc.). Second, qPCR was carried out with a real-time PCR system (Step One Plus, Applied Biosystems) using Eva Green 2× qPCR MasterMix-ROX (MasterMix-R, Applied Biological Materials Inc.). The volume of the qPCR reaction was 10 µL, containing 5 µL of Eva Green 2× qPCR MasterMix-ROX, 0.3 µM forward primer and reverse primer (Table 3), and 1 µL of diluted cDNA (10 ng/µL). The qPCR conditions were 95 °C for 20 s, followed by 40 cycles of 95 °C for 15 s and 60 °C for 30 s. Finally, the specificity products of qPCR were examined by the melting curve. Glyceraldehyde-3-phosphate dehydrogenase (*GADPH*) was used to normalize and calculate the expression of each gene using the comparative threshold cycle method (2^−∆∆CT^).

### 4.8. Statistical Analysis

There were three biological replicates for all the experiments, and the data were expressed as mean ± SD. GraphPad Prism was used for plotting, and the data were analyzed with SPSS statistics and tested by one-way ANOVA. *p* < 0.05 was considered to indicate a statistically significant difference.

## 5. Conclusions

In conclusion, exopolysaccharides from *Chlorella* sp. were extracted and characterized, and the immune activity of CEP4 on RAW264.7 macrophages was investigated. CEP4 can play an influential immunomodulatory role and promote NO, TNF-α, and IL-6 secretion in macrophages. Furthermore, transcriptome analysis revealed that the main function of differentially expressed genes is a biological process (BP), and KEGG enrichment analysis yielded 19 significantly enriched signaling pathways. The results showed that CEP4 could activate the immune activity of macrophages, and the RNA-seq preliminarily revealed the mechanism of action of CEP4 on the immune regulation of macrophages. Finally, the relationship between CEP4 and hub genes and the key targets of CEP4 binding to RAW264.7 macrophages still needs to be further studied.

## Figures and Tables

**Table 1:** Monosaccharide composition.

Name	RT	Ratio (%)
Rha	12.667	3.7
Ara	14.125	8.3
GlcN	16.134	40.8
Gal	18.342	2.6
GlcNAc	21.55	7.7
Xyl	22.65	8.6
GlcA	48.259	21.0

**Table 2:** Top 10 hub genes from the PPI interaction network according to statistics.

Name	Degree	Closeness	Betweenness
*Rbx1*	35	83.66667	1528.84905
*Psma7*	33	80	389.23909
*Snrpd2*	38	82.75	1011.15539
*Nedd8*	40	83.58333	475.38283
*Rps8*	42	84.58333	485.96803
*Rpsa*	40	82.83333	588.27646
*Psmb3*	27	77.5	424.93198
*Isg15*	43	83.58333	1049.77487
*Hsp90ab1*	26	74.08333	1819.91847
*Rps7*	41	84.41667	383.42163

**Table 3:** Mouse primer sequences used for qPCR.

Gene name	Forward Primer (5′→3′)	Reverse Primer (5′→3′)
*GAPDH* [69]	TGCGACTTCAACAGCAACTC	ATGTAGGCCATGAGGTCCAC
*TNF* [69]	ACAAGGCTGCCCCGACTAC	TCTCCTGGTATGAGATAGCA
*NFKBIA* [70]	GAGACTCGTTCCTGCACTTG	AAGTGGAGTGGAGTCTGCTG
*CD14* [71]	GCCAAATTGGTCGAACAAGC	CCATGGTCGGTAGATTCTGAAAGT
*CCL3* [70]	GCCATATGGAGCTGACACC	TTCTCTTAGTCAGGAAAATGACAC
*TGFBR2* [72]	CCGCTGCATATCGTCCTGTG	AGTGGATGGATGGTCCTATTACA
*CTNNB1* [73]	AAGGTAGAGTGATGAAAGTTGTT	CACCATGTCCTCTGTCTATTC
*ITGA4* [74]	TGTGCAAATGTACACTCTCTTCTCCA	CTCCCTCAAGATGATAAGTTGTTCAA
*MT-CYTB* [75]	TGCTTTGAGGTATGAAGGAAAGG	ACATACTAGGAGACCCAGACAAC
